# Challenges in child and adolescent health across the Arab Gulf countries—Focus on metabolic health

**DOI:** 10.1111/cob.70027

**Published:** 2025-06-04

**Authors:** Basma Haris, Madeeha Kamal, Sadriya Alkohji, Shahrad Taheri, Khalid Hussain

**Affiliations:** ^1^ Division of Endocrinology, Department of Paediatrics Sidra Medicine Doha Qatar; ^2^ Division of Adolescent Medicine, Department of Paediatrics Sidra Medicine Doha Qatar; ^3^ Department of Medicine for Child and Adolescent Health Ministry of Public Health Doha Qatar; ^4^ Department of Medicine Weil Cornell Medicine‐Qatar Doha Qatar; ^5^ National Obesity Treatment Centre, Qatar Metabolic Institute Hamad Medical Corporation Doha Qatar

**Keywords:** child metabolic health, paediatric diabetes mellitus, paediatric obesity

## Abstract

The Gulf Cooperation Council (GCC) consists of six member states (Bahrain, Kuwait, Oman, Qatar Saudi Arabia and the United Arab Emirates). The combination of an increasing youth population combined with rapid modernisation, increasing per capita income and the paradigm shift towards unhealthy and affluent lifestyles has created a significant burden of non‐communicable diseases (NCDs), especially obesity and diabetes. The aim of this review is to discuss the prevalence of childhood/adolescent obesity and diabetes in the GCC region, which are some of the highest in the world. The review also focuses on the major challenges the GCC region faced in dealing with these lifestyle‐related NCDs. Children and adolescents lack access to specialised professionals for disease management across all healthcare services in the region, with a lack of multidisciplinary teams and support groups. The development of culturally acceptable and community‐based strategies to promote healthy lifestyles at home and in schools will be essential to achieve sustainable change in these countries to reduce the health and economic burden of metabolic health diseases in children and adolescents in the region.

## 1 INTRODUCTION

The Gulf Cooperation Council (GCC) was established in 1981 and consists of six Arab member states (Bahrain, Kuwait, Oman, Qatar, Saudi Arabia and the United Arab Emirates) bordering the Gulf in the Middle East. The goals of the GCC alliance are political and economic collaboration based on shared objectives built around geopolitical, cultural, religious and racial similarities. The discovery of oil and natural gas, followed by political independence, has transformed the GCC countries into economically strong states with high growth domestic product (GDP) per capita enabling rapid growth and modernisation. Whilst these countries share many similarities, they are different in terms of geographical and population size, governance structure, dependence on expatriate workforce and public and private healthcare service provision. The extent of urbanisation is also different for the different GCC member countries, with the United Arab Emirates, Qatar and Kuwait being the most developed in comparison with the other nations.^1^ Nevertheless, issues like gender roles, inequality and social stigmas are shared across all these nations.

The GCC has one of the fastest‐growing populations in the world. In 2022, the GCC population was estimated to be around 59 million people.^2^ The vast majority of people in the GCC are aged under 25 years. Life expectancy in the GCC countries has increased from 45 to 60 years in 1960 to 76 to 82 years in 2020.^1^, ^3^ The health and quality of life of the GCC population, however, are now increasingly threatened by the rising prevalence of non‐communicable diseases (NCDs) such as obesity, type 2 diabetes mellitus (T2DM), cardiovascular disease (CVD) and mental health disorders.

The GCC countries rank amongst the highest in the world on risk factors related to NCDs amongst adults.^4^ According to the International Diabetes Federation (IDF), the GCC region has six of the 10 countries in the world with the highest adult T2DM prevalence.^4^ Saudi Arabia has the highest prevalence of adult T2DM (20.5%) followed by Qatar (16.3%), Kuwait (17.9%), Bahrain (17.5%), United Arab Emirates (10.7%) and Oman (8.2%).^4^ Also, mortality in the GCC from these NCDs such as diabetes is one of the highest in the world.^5^ The diabetes death rate was 12.1 per 100 000 population in 1990, which increased by 60.7% to 19.5 per 100 000 population in 2013. The rates of diabetes mellitus (DM) in the Middle East region were 1397.6 (age standardised) in comparison to the global estimate of 844.8.^5^ The increased prevalence and mortality of NCDs are primarily due to unhealthy lifestyles, lack of focus on health prevention and disease management, poorly developed primary healthcare provision and inadequate treatment options to manage NCDs and their complications.^5^, ^6^

Children and adolescents (0–24 years of age) comprise more than 50% of the total population of the GCC countries. It is estimated that the number of children and adolescents will be about 38 million in the GCC by the Year 2050.^7^, ^8^ Children and adolescents will have specific healthcare needs based on their developmental stage and individual life circumstances.^9^ Given the shift in lifestyles and household incomes, NCDs are now also the major health‐related issues in children and adolescents in the GCC region.^5^ The health problems and health‐related behaviours arising during childhood and adolescence shape adult health, with important implications for public health.

In this review, we discuss, first, the healthcare needs of children and adolescents in the GCC region with an emphasis on the common non‐communicable metabolic diseases such as obesity and T2DM; second, we describe the scale of the problem for each of these non‐communicable metabolic diseases and then discuss the challenges that are faced specifically by children and adolescents in the GCC states; finally, we review the potential culturally acceptable treatment options and provide guidance for further research specific to the GCC region.

National epidemiological and molecular data on paediatric and adolescent DM are scarce in GCC countries and have focussed mostly on retrospective reviews, questionnaire‐based studies and case reports. If left unchallenged, the consequences of the increasing incidence of DM on the overall health and quality of life of the young generation as well as the economic and manpower burden on the healthcare sector will be profound.

## 2 BURDEN OF METABOLIC DISEASES IN CHILDREN IN GCC COUNTRIES

### 2.1 Childhood obesity–background and scale of the problem

Childhood obesity is one of the greatest health challenges worldwide,^10^ associated with multiple biological, psychological and social consequences that negatively impact quality of life and longevity.^11^ Childhood, and in particular adolescent, obesity tracks into adulthood with further negative consequences for the affected individuals and their families, healthcare services and the economy. GCC countries are perceived to have populations with a high prevalence of obesity in adults and children, making obesity a key area to be addressed through public health measures and clinical service provision. The current prevalence of obesity amongst children in the GCC countries, however, is not accurately known due to variations in population representativeness in studies, disparate non‐standardised measures of obesity including self‐reported values, age groups studied, dates of study conduct and study quality.^12^, ^13^, ^14^ Furthermore, few studies have examined the utility of body mass index (BMI) measure as a reflection of excess adiposity or progression to obesity‐related complications amongst children in the GCC population. BMI underestimates the degree of adiposity when measured and is even a poorer indicator of adiposity when self‐reported measures are employed.^15^

**FIGURE 1:**
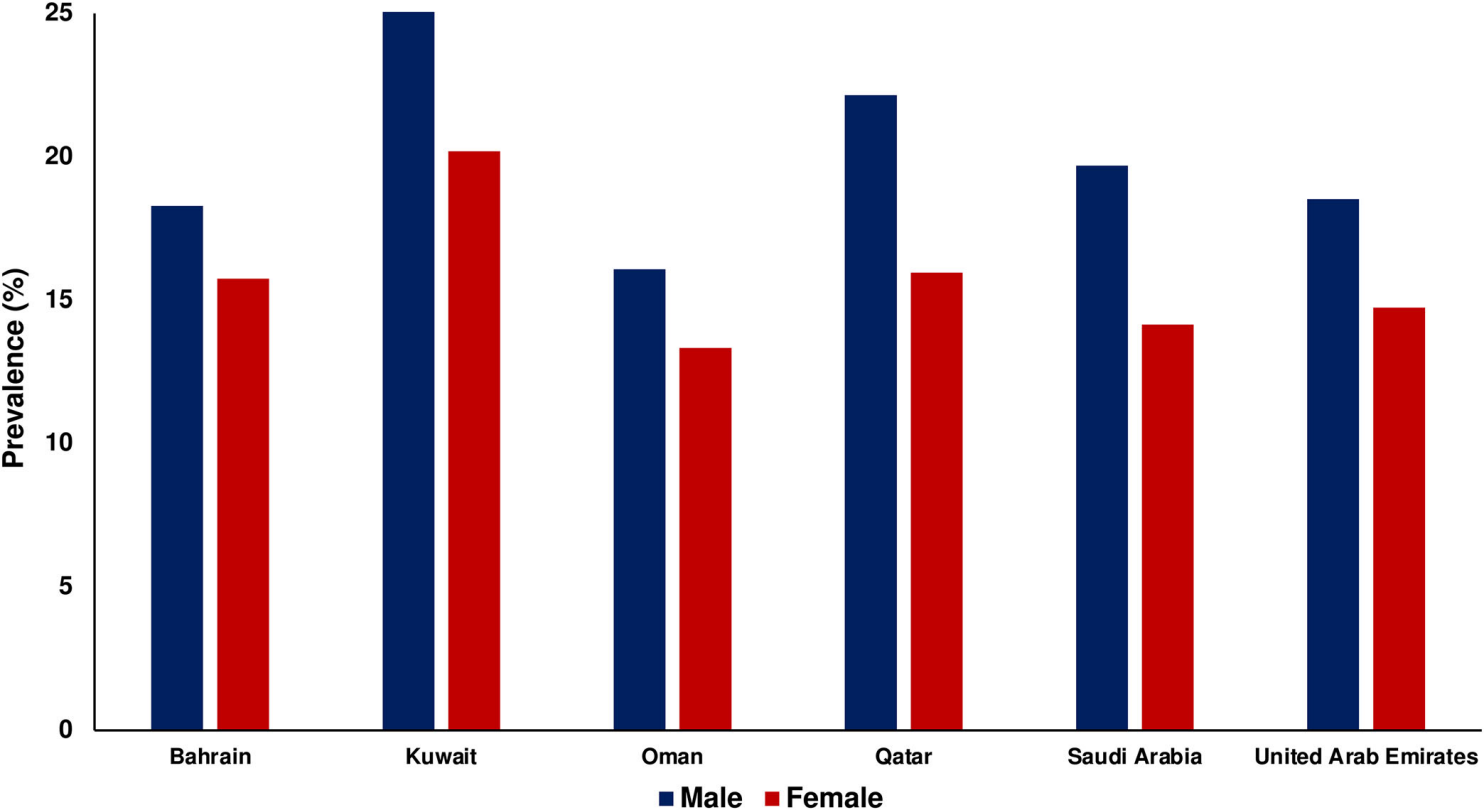
The prevalence of obesity in children in Gulf Cooperation Council countries. Data from the Global Obesity Observatory.

Data from the Global Obesity Observatory regarding the prevalence of obesity amongst GCC are shown in Figure 1. Kuwait and Qatar appear to have the highest obesity prevalence amongst children. A cross‐sectional study of 6574 school children aged 6–18 years from 244 schools in Kuwait (2015–2018) reported the prevalence of obesity across the entire study population as 33.9% using Centers for Disease Control and Prevention (CDC) criteria, 28.2% using World Health Organisation (WHO) criteria and 30.5% using International Obesity Task Force (IOTF) criteria.^16^ The Kuwait Nutrition Surveillance System data of 4400 children aged 2–5 years (2016–2019) showed fairly stable obesity prevalence across the survey years, and the prevalence of obesity was 10.8% amongst those 2 years of age compared to 13.5% amongst 5‐year‐olds using the WHO criteria, a lower prevalence than the previous study from Kuwait.^17^ The largest study from Kuwait (*n* = 172 603) included 5‐ to 19‐year‐olds and reported an overall prevalence of obesity of 28.4% in 2019, compared to 21.4% in 2007.^18^ A stabilisation of the prevalence of obesity was observed in females. The Qatar school childhood measurement programme reported the prevalence of obesity in children aged 5–19 years measured in 2015–2016.^19^

Thus, about one in five to one in three children in Kuwait and Qatar may be classified with obesity, a prevalence similar to the United States. The largest study from Saudi Arabia, the most populous GCC country, is the School‐Based Screening Program that included 444 259 school children aged 6–14 years (2018–2019).^20^ This study reported an obesity prevalence of 4.1% using BMI data plotted on Saudi Arabian sex‐specific percentile charts, a prevalence that is significantly lower than other smaller regional reports from Saudi Arabia that used international BMI cut‐points for obesity, such as the WHO criteria, which estimated obesity prevalence at over 10%. The prevalence of obesity reported from the GCC countries is higher amongst adolescents and boys. The gender difference in obesity is opposite in adults, where there is a greater prevalence in women. These differences in the epidemiology of obesity in children and adolescents in the GCC can partially be explained by the different methods used to collect the data. For example, some of the studies were surveys only, some involved retrospective data collection, whereas others were prospective. In addition, individual risk factors such as the ethnic background of the child, dietary habits, physical activity, family educational level and income, but also regional differences in access to health care, availability and easy access to physical activities will also account for the variablity.^21^ The prediction of the future prevalence of obesity is hampered by inconsistent non‐standardised measures across GCC countries, and there is likely to be an impact of the prolonged Corona Virus Infektion (COVID‐19) pandemic and the lockdown that created a milieu for unhealthy lifestyles by forcing children to stay at home and not partake in school or sports activities. Hence, the incidence of obesity and DM downstream is expected to rise further. Insulin resistance is a common complication of obesity which leads to prediabetes, and annually 5%–10% of those with prediabetes progress to T2DM.^22^, ^23^

The two GCC countries with the highest estimated prevalence of obesity in children (Kuwait and Qatar) have a higher GDP per capita ($24 809 and $50 815, respectively) compared to the GCC countries with the lowest estimated prevalence (Bahrain $19 925 and Oman $12 409).^24^ Amongst GCC countries, obesity is a disease of affluence unlike western countries where it is more associated with poverty and social deprivation. Whilst GCC countries are currently in the top 100 countries for GDP per capita (highest ranked is Qatar at 17th, and lowest Oman at 74th),^24^ the potential economic costs of obesity will become unsustainable over the next few decades, especially with the global trend towards sustainable energy sources and climate action, thus diminishing revenue from non‐renewable energy sources on which GCC countries depend heavily. This will lead to budget deficits and potential risk to the stability of the governments. This can affect individuals and businesses as well since the governments may be forced to take steps to compensate for the deficit by reducing funding for healthcare and research or increasing taxes. Based on obesity NCD preparedness country rankings from the World Obesity Atlas 2022, Oman (49th), Bahrain (54th), Qatar (62nd) and Kuwait (72nd) are in the group of countries that appear to be better prepared in the Eastern Mediterranean region. However, these rankings are likely to change based on economic development and growth. It is important to note that GCC countries spend only about 5% of their GDP on healthcare, a figure significantly lower than the United Kingdom (12%) and the United States (19%).

### 2.2 Childhood diabetes mellitus—background and scale of the problem

DM is one of the most common chronic metabolic disorders in children and adolescents. DM is already a major global public health burden and the IDF Diabetes Atlas reports 1.1 million individuals below 20 years of age with type 1 diabetes.^25^ Recent data also estimate an average increase of 3%–4% in the prevalence of DM in the world per year.^26^ The financial cost of dealing with DM poses a significant burden on the health budgets of different countries. For instance, Saudi Arabia spent $12bn in 2015 on DM management whilst Qatar, Bahrain and United Arab Emirates spent $0.5bn, $0.27bn and $2bn, respectively, on tackling DM in 2015.^25^, ^27^, ^28^ This is not considering individuals with prediabetes and the individual, family, community and social impact of the condition, sick days at school, early complications and disability, provision of necessary training to healthcare providers amongst other unquantifiable factors.

The estimated incidence rate of type 1 diabetes in children was 25.1 per 1000 children in Europe when compared to the Middle East and North Africa (MENA) region, where it was 14.4 per 1000 children.^27^ Saudi Arabia and Kuwait have reported an incidence of 31.4 and 22.3 per 100 000, respectively.^27^, ^29^ A prospective countrywide study in Qatar reported the incidence of all types of DM in children as 39.19 per 100 000 in 2020 and a prevalence of 301.91, thus placing Qatar amongst the top five countries in the world for the incidence of DM in children.^30^ The same study reported the incidence of type 1 diabetes as 38.05 per 100 000 in 2020.^30^ Reported incidence from Oman and Kuwait was 2.7 and 44.5 per 100 000 people, respectively.^31^ The incidence and prevalence values reported by different studies in the same country vary widely, making accurate analysis difficult. There appears to be an increase in the overall incidence of type 1 diabetes in the GCC region when compared to previous reports, along with a lowering age of onset of diabetes.^26^, ^32^

With the increasing prevalence of obesity in children, the incidence of T2DM is rising.^33^ The incidence of T2DM was 2.51 per 100 000 in the paediatric population in Qatar.^30^ whilst a study from Kuwait reported a prevalence of T2DM as 33.2 per 100 000 in children.^34^ The data indicate that the GCC countries, and Qatar in particular, have a higher prevalence of T2DM in children when compared to their western counterparts and similar to that observed in adults. Table 1 shows the epidemiology of paediatric DM in GCC countries.

**TABLE 1:** The available epidemiology of paediatric diabetes mellitus in Gulf Cooperation Council countries.

Country	T1DM incidence	Number of children	T2DM prevalence
Bahrain^29^	2.7	96	‐
Kuwait^29^	44.5	5496	33.2 per 100 000
Saudi Arabia^6^, ^28^	31.4	28 900	9.04%
Oman^29^	2.7 (6)	355	‐
Qatar^28^	38.05	1325	23.7 per 100 000
United Arab Emirates^29^	‐	‐	11%

Abbreviations: T2DM, type 2 diabetes mellitus; T1DM, type 1 diabetes.

## 3 REASONS BEHIND TRENDS

A person's behaviour is influenced by many factors including individual's relationships with others, community, job, school, family, their lifestyle, genetics and the laws that the government has put in place (ecosystem). In relation to obesity, the key individual drivers in the GCC region are culture and lifestyle, environmental factors and possibly genetic factors. These will be reviewed below in detail.

### 3.1 Cultural and lifestyle factors

The increasing prevalence of metabolic diseases in the region is multifactorial. The reported high prevalence of childhood obesity within the GCC population has occurred simultaneously with rapid modernisation. This unprecedented rapid transformation has been accompanied by a nutrition transition from the traditional Mediterranean‐style diet with limited portions due to scarcity to a more western‐type diet characterised by refined grains, low fruit and vegetable intake, and accompanied by increased intake of processed, ultraprocessed and fast foods and sugar‐sweetened beverages that are readily available and delivered to the home at any hour.^35^, ^36^, ^37^, ^38^, ^39^, ^40^ Also, diverse expatriate populations within GCC countries have introduced the indigenous populations to a wide array of international cuisines. Breakfast skipping has been observed to be associated with obesity in the GCC paediatric population.^41^ Food and high energy drinks play a central role in social interaction and hospitality amongst the GCC population, and children are frequently offered food by family and friends, resulting in energy overconsumption. Also, buffet style eating is common, with multiple varieties of food from different cuisines, fast food and high energy processed snacks provided together throughout the day.^42^

Levels of sedentariness and low physical activity are particularly high amongst children and adults in GCC countries. GCC countries score low on the physical activity report card.^43^, ^44^ In a study including children aged between 14 and 19 years from Saudi Arabia and Kuwait, it was found that 50% of boys and 70% of girls do not meet the minimum daily physical activity criteria of 1 h set forth by the WHO.^45^ School‐based health surveys from Qatar, United Arab Emirates and Oman have also reported similar findings.^46^ A cross‐sectional study in 2017 of 1053 children from Oman with an average age of about 9 years using a uniaxial accelerometer reported that only about 40% achieved 60 min of moderate to vigorous activity across the day.^47^

In the United Arab Emirates, a cross‐sectional study of 133 (mean age 10.5 years) children observed that 19% had accelerometry‐measured daily moderate to vigorous physical activity at school.^48^ In a study from Qatar, including 5682 adolescents, 35% reported being active for 60 min on 3 days/week.^49^ Most studies regarding physical activity from GCC countries have used self‐reported data and have not included representative samples. It is crucial to emphasise and acknowledge that the GCC states generally lack population surveillance of physical activity and sedentary behaviours across the life course, and the cited evidence is not from rigorous surveillance, which is widely available in other high‐income countries.

Screen time amongst children is believed to be a contributory factor to sedentary behaviours and unhealthy food intake amongst children in GCC countries.^14^, ^50^, ^51^, ^52^, ^53^ Short sleep duration and irregular sleeping habits have also been observed to be associated with obesity amongst the paediatric population. In a study of 385 schoolchildren (aged 7–12 years), the average actigraphy‐measured sleep duration was below the recommended duration for this age group and sleeping less than <8 h was associated with an increased risk of obesity.^54^

### 3.2 Changes in policy

Reduced growth in sales of sugar‐sweetened beverages has been observed in some GCC countries, particularly in Saudi Arabia, where this taxation was introduced first.^55^ The long‐term impact of these efforts on obesity remains to be determined. Several countries are considering restrictions on advertising for unhealthy foods. Harnessing social media and the internet will be crucial for the success of campaigns to prevent obesity in GCC countries.^56^ Nutrition guidelines have been produced by several GCC countries and there is a movement towards providing more standardised nutrition labels on purchased foods, but most guidelines reflect western nutrition guidelines, food labelling is moving forward at a slow pace, and there are little concerted continuous campaigns to educate the population regarding nutrition.

### 3.3 Environmental factors

The environment in most GCC countries is dominated by high outside temperatures, particularly in the summer months, which limits outdoor activity. There is high reliance on cars for transportation, even for small journeys, and insufficient provision for safe walking space and public walkways.

### 3.4 Genetic factors

Monogenic and syndromic obesity are not notably more frequent in GCC countries and there are only a few unique genetic polymorphisms of undetermined significance linked to obesity in the Arab population.^57^ Given the high prevalence of obesity in women of about 40% in GCC countries, maternal obesity, gestational weight gain and gestational diabetes mellitus (GDM), through the intra‐uterine environment, could be key contributors to obesity in childhood and beyond.^58^ In a systematic review, the prevalence of GDM in Saudi Arabia was 17.6%, Qatar 14.7% and Bahrain 12.2%.^59^ Data from the IDF, however, show a higher standardised prevalence of GDM for the Middle East of 27.6%, the highest of all regions; in comparison, the standardised prevalence for North America and Europe were 7.8% and 7.1%, respectively.^60^

## 4 CHALLENGES AND FUTURE INITIATIVES

### 4.1 Healthcare access and quality

Currently, there are few specific medical centres in GCC countries catering for children with obesity and diabetes. The lack of primary healthcare services and providers trained in specifically adolescent health leads to teens being cared for by physicians for adults. This runs the risk of adolescents not being diagnosed accurately in a timely manner and managed appropriately.^61^ The fact that the great majority of teenagers go untreated or are treated incorrectly also has an impact on the accuracy of epidemiological data gathering.^62^ The reason behind this is not only related to the training provider but also due to cultural factors like teens not wanting to be treated by a ‘children's doctor.’ There is also a paucity of clinical trials data on this population since doing clinical trials for new medications has proven challenging due to cultural reasons and over‐legislation. Research trials are still considered a novelty and insignificant and sometimes even with distrust culturally, with families avoiding hospitals where they may be asked to take part in a research study. This has also led to over‐legislation from governments which discourage healthcare providers from undertaking trials.^63^

Many governments, including those in GCC countries, have continued to frame obesity as a problem arising from individual choices. Obesity, however, is a complex phenomenon and a key element in tackling it is collaboration across governmental structures and private groups to coordinate an action plan for obesity. The World Obesity Federation Strategic Plan 2020–2025 has set forth certain global goals and targets, such as zero increase in adult and childhood obesity prevalence by 2025 in comparison to the WHO targets in 2013 and 2015, one‐third reduction in premature NCD mortality by 2030, and to end malnutrition in all its forms. Policy recommendations from global frameworks such as the WHO Commission on Ending Childhood Obesity (ECHO) include calls to address marketing to children, advance physical activity and deliver school‐based programmes, amongst other strategies.^64^ The Open Policy Engagement Network (OPEN) Gulf is a novel initiative aiming to develop policies for tackling obesity in the GCC countries through a multi‐sectoral approach.^65^ It is envisaged that the effort will allow each GCC country to develop and extend individual country‐specific policies whilst adopting best practices through knowledge exchange. Part of this effort is to ensure that children with obesity are provided with adequate access to appropriate medical care. As the burden of obesity and NCDs increases in the GCC countries, a greater proportion of GDP will need to be spent on health when revenue from natural gas and oil declines.

**FIGURE 2:**
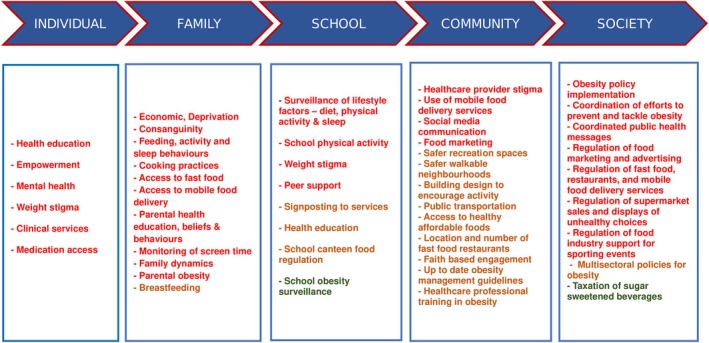
The levels of achievement across potential initiatives for tackling obesity in Gulf Cooperation Council countries. The achievement is colour coded red (not addressed), orange (partially addressed) and green (addressed).

Figure 2 shows the levels of achievement across potential initiatives for tackling obesity in GCC countries. The limited accurate data and lack of established surveillance for obesity suggest that insufficient priority is given to childhood obesity by GCC countries. Investment is required to conduct rigorous, coordinated, standardised and periodic surveillance of obesity and diabetes as well as its contributing factors, such as nutrition, physical activity levels and sleep patterns, duration and quality amongst children. The adoption of school measurement programmes across several GCC countries should facilitate closer monitoring of obesity trends and assessment of the impact of any public health measures to reduce obesity across GCC countries. A Social Ecological Model (SEM) is a commonly used model in childhood obesity research and prevention initiatives, which takes into account concentric layers of influence encompassing intrapersonal, interpersonal, community, organisation, government, industry and societal domains. Community‐level factors like school awareness programmes, particularly for older children, for whom school environments may have a greater influence, physical activity facilities as well as child‐level factors like age, race and gender; parental characteristics like weight status and education level could be couched together in the SEM. When opportunities arise, such a system will support the development of cross‐level connections and solidify existing ones.^66^

The management of obesity in the GCC, especially in adolescents, has been dominated by bariatric surgery.^67^ The availability of high disposable income, social media influence and burgeoning private healthcare has resulted in many members of the same family, across all age groups, receiving bariatric surgery, many without fulfilling internationally accepted eligibility criteria. Furthermore, patient follow‐up post‐bariatric surgery is inadequate, resulting in consequences of multiple nutritional deficiencies and weight regain.^67^ The GCC countries need to plan for coordinated management of children and adolescents with obesity and provide multidisciplinary and multi‐professional services for obesity management. These services should provide evidence‐based treatments and follow‐up, including lifestyle and psychological support, pharmacotherapy, endoscopic procedures and bariatric surgery for appropriately selected patients.

### 4.2 Public health and prevention

Another challenge in GCC countries is that obesity in children is still perceived to be an indicator of optimal health and family wealth. The care of children is frequently delegated to expatriate caregivers in many GCC countries. The extended family has an important role to play in determining food quantity and quality and physical activity. Thus, health education interventions to tackle childhood obesity need to include parents, the extended family and carers. Concerted efforts should be directed at implementing education campaigns aimed at parents and the public and identifying effective approaches for obesity prevention that are tailored to the GCC population. The high incidence of diabetes in the GCC often results from a lack of appreciation for the seriousness of diabetes since many family members are afflicted by diabetes, thus its impact is lessened.

Access to fresh fruit and vegetables has been a challenge for several GCC countries which have relied mostly on imported food. To encourage healthy food intake, GCC countries have developed nutrition guidelines, but adherence to the guidelines has been challenging.^13^ Efforts are underway to improve greater food self‐sufficiency in the GCC countries. Programmes have been initiated to deliver public health and school campaigns^68^ to encourage healthy eating and physical activity, to provide guidance on food provided in school canteens, and for taxation of sugar‐sweetened beverages (implemented from 2017 by three GCC counties with others joining in 2019). The development of comprehensive programmes aimed at the implementation of the guidelines outlined in the WHO Ending Childhood Obesity Report 2016^69^ with the help of non‐governmental organisations, private sector and academic institutions could prove to be of great value.

Parental obesity is associated with obesity in children, thus creating a cycle of obesity that is perpetuated across generations. Targeting maternal obesity and improving breastfeeding are potential strategies that might be effective in efforts at reducing the burden of childhood obesity in GCC countries.^70^ Several GCC countries are now targeting maternal obesity and gestational diabetes and putting efforts into encouraging breastfeeding initiation and continuation.

Studies in adults suggest that T2DM, if diagnosed early, can be reversed or at the very least managed without further progression to complications with lifestyle interventions.^71^ These include a healthy diet, physical activity, weight loss along with regular medical reviews and tight glycaemic control. Early screening to identify at‐risk individuals, like close family members, can be implemented at a nationwide level to improve surveillance. Long‐term follow‐up of individuals with prediabetes along with lifestyle interventions has proven to be effective in preventing the metabolic syndrome in 26% of the study group in a study from Saudi Arabia.^72^ Multidisciplinary committees for intensifying health promotion activities to raise awareness about childhood obesity and the diabetes epidemic and its prevention are lacking in some GCC countries like Kuwait and Oman. The development of such teams could help with the development of campaigns to promote healthy environments in malls and other public areas, increasing physical education at schools and working with supermarkets and school canteens to endorse healthy eating amongst children and youth. Another potential way to curb unhealthy diet habits is to ban the marketing of fast food and sugar‐sweetened beverages in children's media channels and levy more taxes on such establishments. Taxation on sugar‐sweetened beverages has been implemented in some of the countries in the GCC, such as the United Arab Emirates, Bahrain and Saudi Arabia, and it has resulted in a drop in the growth of sales in these countries.^73^ This is expected to also result in a reduction in the prevalence of metabolic diseases; however, other areas such as the advertising of unhealthy food, proximity to and accessibility of fast food restaurants have not been addressed.

## 5 PLANNED PROGRAMMS FOR THE FUTURE—LIMITATIONS AND GOALS

Many GCC countries have implemented various programmes for better surveillance and management of children with DM. In Qatar, the Qatar Foundation and Qatar National Research Fund have supported many projects directed towards paediatric DM, but these are research funded rather than directly implemented through government public surveillance action. In Saudi Arabia, an adolescent medical task force was constituted in 2007 for the development of specialised adolescent health and medical centres.^71^ The Dubai Diabetes Center in United Arab Emirates, the Dasman Diabetes Institute in Kuwait and Bahrain Diabetes Centre were constituted to focus on research initiatives as well as providing support to children with diabetes.^4^ Although a considerable amount of budget has been allocated towards research and development, precision medicine and medical technology, the availability is proportionately lower when compared to the demand. There is a deficiency of locally trained healthcare workforce (physicians, nurse DM educators and dieticians) who understand the culture, and the healthcare needs are mainly dependent on expat expertise which changes rapidly over time. The GCC countries need to invest in the local workforce in the long term; however, this might be a challenge for the smaller GCC countries that have very small indigenous populations. Therefore, there needs to be a drive to integrate expatriates into these countries and provide them with sufficient rights to maintain a stable workforce in order to respond to the current issues faced by the countries.

There is a huge potential and need for the development of a common childhood diabetes centre in the region, even if only virtually. Such a centre could function as an academic centre with research links as well for the development of novel therapies and trials as well as elucidating the molecular mechanisms behind the high prevalence of paediatric DM observed in the GCC population. However, currently there is poor collaboration between GCC states in DM research. Collaboration and knowledge exchange between GCC states need to be encouraged. The development of a paediatric diabetes registry for the whole region or in each country individually, where every child with DM is entered into the registry, will be very useful to get accurate epidemiological data and bring more focus to the DM epidemic in the region.

Adolescents with DM have specific social and psychological needs which are not always met in all the GCC states and there is a lack of organised diabetes transition services from adolescents to adults. This transition needs to be planned by well‐trained medical personnel and legislators to facilitate a seamless transition keeping in mind the specific needs of this age group.

More specialised training opportunities can be developed to educate more scientists specialising in beta‐cell biology and induced pluripotent stem cell research. Potential new treatments involving islet cell transplantation, stem cell‐derived beta‐cell replacement are on the horizon, offering a potential cure for type 1 diabetes.^74^ Dual acting and triple acting incretin mimetics are also in development, which show promise in clinical trials not only in the treatment of T2DM, but also for other obesity‐related complications.^75^ Yet, many novel treatments take longer to be introduced in the GCC countries, and some are freely available over the counter without appropriate medical oversight. Insulin pump therapy is becoming increasingly popular in the GCC countries, with studies reporting good glycaemic control and reduced occurrence of hypoglycemia.^76^ There should be a readiness in the GCC countries to embrace these new treatments and collaboration with research institutes for clinical trials in the region.

Many of the GCC countries have now prioritised NCDs‐related health problems in their national healthcare strategies. All GCC countries have adopted the WHO health city initiative to ensure the creation of synergistic efforts between different sectors affecting health determinants such as education, agriculture, environment and transport to promote the well‐being of children and adolescents and their families.

The future plan should be to continue to build an acceptable and responsive health system in which the population has confidence across all the environments where children live, grow, learn and play: to link sectors to enable and address the wider determinants of health, whereby services are integrated with a focus on continuity and coordination across all levels of physical health and social care. There is a particular need to ensure as we reset and recover from the impact of COVID‐19 that the lessons learnt are acted on and that services are developed equitably.

The planned programmes can help inform policy makers to develop the supportive measures to aid lifestyle modifications in the population. However, they also require the commitment of non‐health sectors, which is a challenge since they may not prioritise these measures in their strategic planning and initiatives. Addressing NCDs, therefore, effectively demands a ‘whole‐of‐government,’ ‘whole‐of‐society’ and ‘health‐in‐all policies’ approach. It is also important to consider following specific principles in improving healthcare system targeting NCDs amongst children and adolescents:

- Establish child‐centred interventions in dealing with the underlying social determinants relevant to NCDs. A child‐centred approach involves engaging with and listening to what children and young people have to say about what is happening to them and this should be the cornerstone of effective intervention and support. Social determinants play a role in shaping risk factors for NCDs such as obesity and diabetes and efforts to improve social determinants of health, such as early childhood education and parenting skills, education and lifelong learning, working and employment conditions, poverty reduction and ensuring a healthy standard of living, housing and the environment and prevention of ill health will all help to reduce the burden of diabetes and obesity.
- Establishment of funding guidance to support collaborative action to prevent, diagnose, treat and research NCDs amongst children and adolescents. Funding projects which support the prevention, diagnosis, treatment and research into NCDs amongst children and adolescents are vital in tackling NCDs such as obesity and diabetes. An example of collaborative funding guidance for NCDs (including obesity and diabetes) is the recent (December 2021) launch by the European Commission of the Healthier Together—EU NCDs initiative. This supports EU countries in identifying and implementing effective policies and actions to reduce the burden of major NCDs and improve citizens' health and well‐being.^77^ However, no such collaborative funding exists in the GCC region for action on NCDs amongst children and adolescents.
- Promote community‐based support groups for children and families who are living with NCDs. Community‐based health interventions are a key part in the prevention of NCDs.^78^ The type of community‐based interventions can be at different levels, namely individual, group, community and policy levels, but individual and group level interventions are more frequently used.
- Enhanced initiatives to promote healthy dietary habits and physical activity amongst this age group. Food labelling and dietary and nutritional guidelines will help promote healthy dietary habits. This has been successfully implemented in some countries with a real impact on obesity. For example, in Finland there are recommendations to reduce access to sugary, high‐fat snacks and drinks in school vending machines and rules on how foods can be marketed to children. Sweets, chocolate, soft drinks and ice cream are taxed at higher rates throughout the country. These measures have helped to slow down the obesity epidemic in some regions of Finland.^79^ The physical activity initiatives include creating active environments (access to open spaces, parks, playgrounds and access to appropriate equipment for physical activity), offering choice and variety (offering variety of physical activity, opportunities to take part, including free play), embedding physical activity in the curriculum (increase time spent on physical activity) and promoting active travel.
- Ensure that the national health insurance system includes coverage for NCDs affecting children. Universal health coverage is central to developing policies for tackling NCDs.^80^ Having a national health insurance system will allow access to all essential primary health services for all, ensure equity of health care, allow screening of those at high risk from NCDs, and promote education.
- Identify attainable child‐specific targets and committed monitoring systems to monitor and evaluate child‐specific actions. This may involve setting specific target behaviours on which the child and family can focus and encourage self‐monitoring. Working with the families to set realistic achievable goals with the goals stating the specific behaviours to be targeted.
- Setting realistic and achievable goals is a critical strategy in weight management and should focus on both diet and lifestyle changes. Establishing short‐term specific, measurable, attainable, realistic and timely (SMART) goals is a way to help children succeed. SMART provides the detail, support and guidance children and parents need to stay focused on weight‐loss goals.^81^

## 6 CONCLUSION

The emergence of NCDs such as obesity and diabetes in children and adolescents will have an enormous impact on healthcare globally. In the GCC region, this is further compounded by complex factors such as culture, environment and possible genetics. For the GCC states, the biggest challenges are the development of culturally and environmentally based health promotion strategies targeting the key diseases such as childhood obesity and diabetes. These health promotion strategies need to be implemented so that, for example, healthy lifestyles become the norm for children at home and at school. In addition, there needs to be an evaluation of these strategies to understand the impact on the population. The other major challenge for the GCC states is the availability of nationally trained professionals for health promotion at all levels. These challenges are at the root of developing any practical and consistent healthcare promotion strategies to combat the challenges of obesity and diabetes in children and adolescents and are thus extremely important. The healthcare promotion strategies should be designed to create sustainability and should be implemented as soon as possible, or the GCC states will face an ever‐increasing burden of these NCDs such as obesity and diabetes in children and adolescents in the future.

## AUTHOR CONTRIBUTIONS

**Basma Haris**, **Madeeha Kamal**, **Sadriya Alkohji**, **Shahrad Taheri** collected, analysed and interpreted data, drafted the manuscript. **Khalid Hussain** designed the study, reviewed and edited the manuscript. **Basma Haris** and **Khalid Hussain** integrated all author contributions and produced the first draft of the manuscript, which was then reviewed and critically revised by all authors. All the contributors contributed equally to drafting the manuscript.

## CONFLICT OF INTEREST STATEMENT

No conflict of interest was declared.

## Data Availability

Data sharing is not applicable to this article as no new data were created or analyzed in this study.
